# Improved optical limiting performance of laser-ablation-generated metal nanoparticles due to silica-microsphere-induced local field enhancement

**DOI:** 10.3762/bjnano.6.122

**Published:** 2015-05-22

**Authors:** Zheren Du, Lianwei Chen, Tsung-Sheng Kao, Mengxue Wu, Minghui Hong

**Affiliations:** 1 Department of Electrical and Computer Engineering, National University of Singapore; 2 Engineering Science Program, National University of Singapore; 3 Department of Photonics and Institute of Electro-Optical Engineering, National Chiao Tung University, 1001 Ta hsueh Rd., Hsinchu 30010, Taiwan

**Keywords:** laser ablation, local field enhancement, microspheres, nanoparticles, optical limiting

## Abstract

For practical application, optical limiting materials must exhibit a fast response and a low threshold in order to be used for the protection of the human eye and electro-optical sensors against intense light. Many nanomaterials have been found to exhibit optical limiting properties. Laser ablation offers the possibility of fabricating nanoparticles from a wide range of target materials. For practical use of these materials, their optical limiting performance, including optical limiting threshold and the ability to efficiently attenuate high intensity light, needs to be improved. In this paper, we fabricate nanoparticles of different metals by laser ablation in liquid. We study the optical nonlinear properties of the laser-generated nanoparticle dispersion. Silica microspheres are used to enhance the optical limiting performance of the nanoparticle dispersion. The change in the optical nonlinear properties of the laser-generated nanoparticle dispersion caused by silica microspheres is studied. It is found that the incident laser beam is locally focused by the microspheres, leading to an increased optical nonlinearity of the nanoparticle dispersion.

## Introduction

Laser ablation in liquid (LAL) is a versatile technique to fabricate nanoparticles. Conventional synthesis of nanoparticles by chemical reactions is usually complicated and uses toxic materials. The chemically synthesized nanoparticles are easily contaminated by chemical precursors, additives or byproducts. LAL has many advantages, such as the simple, safe, and high purity properties of the generated nanoparticles, the variety of the target materials capable of being produced, and the in situ dispersion of the nanoparticles. The properties of the laser-generated nanoparticles, such as size, size distribution, composition, and structure for different target materials can be flexibly controlled by the laser parameters (e.g., wavelength, repetition rate, pulse duration, and laser fluence) and the properties of the environment (e.g., surfactant concentration, pH value, or size and length of ligands) [1–6]. Laser ablation is an explosive material removal process using strong pulsed-laser irradiation. The LAL process causes the ejection of nanoclusters into the environment to form a colloidal nanoparticle dispersion [7–9]. The nanoparticles have wide application in physics, chemistry, biology and engineering. One of the important applications is that the nanoparticles can be used as optical limiting materials for the protection of the human eye and in optical devices against high intensity light irradiation.

A material exhibiting the optical limiting effect has a decreased optical transmittance with increasing incident light intensity. A good optical limiter should have high transparency at low light irradiance, while it efficiently attenuates high incident light irradiance. Nonlinear optical material interactions, such as nonlinear absorption, refraction and scattering [10–12], are the main optical processes which contribute to optical limiting. Various materials have been found to exhibit optical limiting properties, such as fullerenes, metallophthalocyanines, carbon black suspension and nanoscale metallic materials [13–15].

For the practical use of the optical limiting materials, it is important to improve their optical limiting performance, including a lower optical limiting threshold and the ability to efficiently attenuate high intensity light. In this paper, we fabricate nanoparticles of different materials by LAL. We investigate the optical nonlinear properties of the laser-ablation-generated nanoparticle dispersion. A novel method is proposed to improve the optical nonlinear properties of the dispersion by coupling with silica microspheres. It is found that the incident laser beam is locally focused by the microspheres, leading to an improved optical nonlinearity of the nanoparticle dispersion. The optical limiting threshold of the optical limiting material is significantly lowered due to the enhanced nonlinear refraction.

## Experimental

### Materials

**Figure 1:**
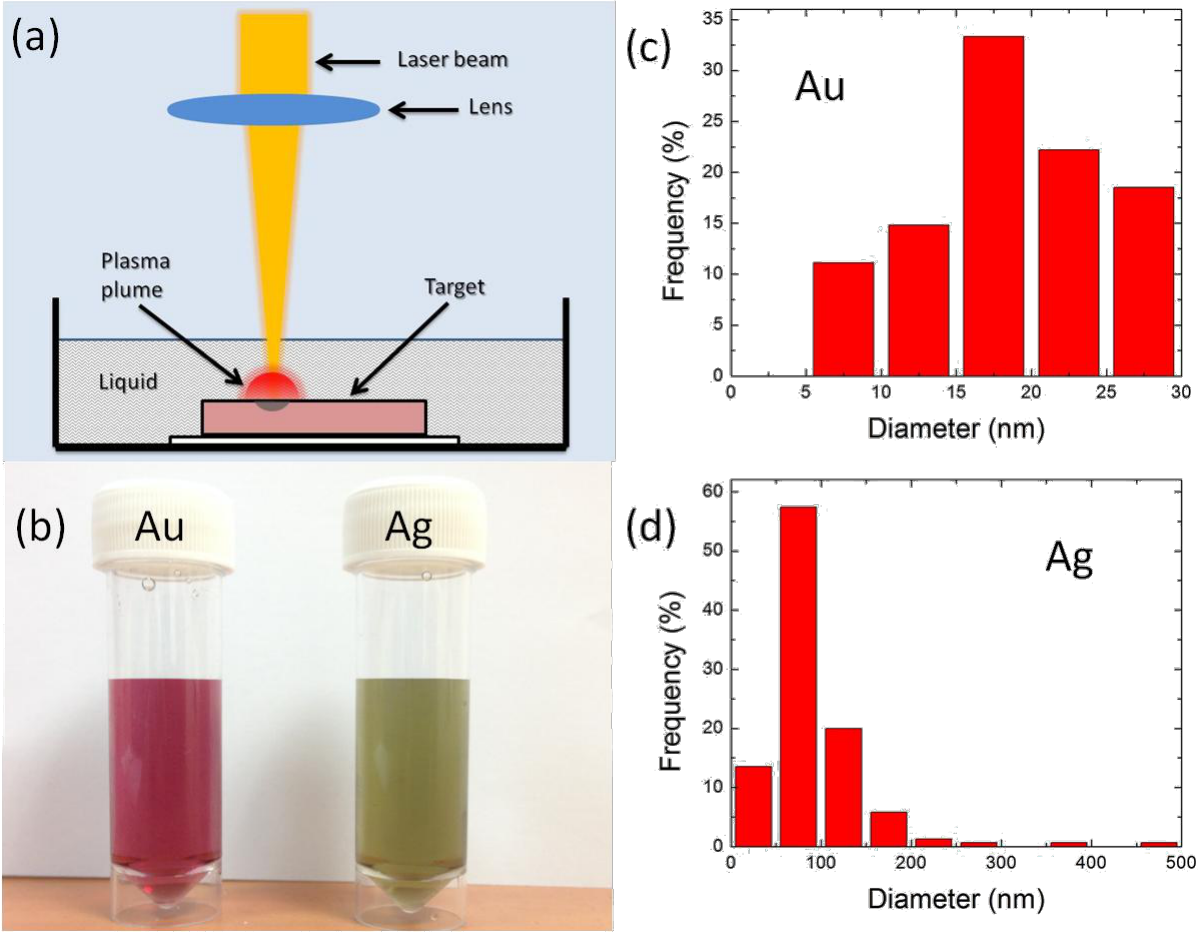
(a) Schematic of the LAL experimental setup. (b) Photograph of laser-generated gold and silver nanoparticle dispersions by the LAL technique. Size distributions of (c) gold and (d) silver nanoparticles estimated from SEM images.

The nanoparticle dispersions were fabricated by the LAL technique as schematically shown in Figure 1a. A solid target material was placed at the bottom of a beaker and immersed in 10 mL of deionized (DI) water. A fiber laser with a wavelength of 1064 nm, a pulse duration of ≈10 ns and a repetition rate of 100 kHz, was focused onto the surface of the target material with a spot size of ≈30 μm. The applied laser fluence was 12.5 J/cm^2^. The laser beam was programmed to scan over a 1 × 1 cm^2^ area 900 times. A square scanning pattern was chosen and the scanning speed was set at 1 mm/s. The entire process took about 5 min. The laser-generated nanoparticles were dispersed into the DI water. The dispersion was collected and sonicated for 30 min to ensure the uniform distribution of the nanoparticles in the DI water. The target materials included gold and silver of high purity. Silica microspheres (purchased from Thermo Scientific) with a diameter of 1 μm were used in this study. The number of microspheres per unit weight was calculated to be 9 × 10^10^ g^−1^. 0.2 mg of microspheres was uniformly dispersed in a 3 mL nanoparticle dispersion to form a hybrid material dispersion.

### Measurement

The laser-generated nanoparticles were characterized by scanning electron microscopy (SEM). The size distribution of the nanoparticles was estimated by analyzing the SEM images. The fiber laser for LAL was also used for the optical limiting measurement. The nanoparticle and hybrid material dispersions were placed in a glass cuvette with a light path of 1 mm for the optical limiting measurement. The incident and transmitted laser powers were measured.

## Results and Discussion

The nanoparticle dispersions of different materials were fabricated by LAL as shown in Figure 1a. LAL is an explosive material removal process that can be used for the synthesis of nanoparticles. The interaction between ns laser pulses and a solid target in a liquid environment typically involves plasma formation, expansion and shock wave generation [16–17]. A high density of energetic species is generated in the plasma plume during the ablation process. The energetic species includes atoms, molecules, ions, clusters, and ultrafine particulates. The strong interaction among these energetic species and liquid molecules promotes nucleation and aggregation, leading to the formation of the nanoparticles [1].

Figure 1b shows a photograph of the laser-generated gold and silver nanoparticle dispersions fabricated by the LAL technique. Droplets of the nanoparticle colloidal solution were placed on a polished silicon substrate. These droplets were dried and the nanoparticles on the silicon substrate were characterized by SEM. The particle size and size distribution of the gold and silver nanoparticles are summarized in Figure 1c,d. This shows that the gold nanoparticle diameter distribution ranges from 5 to 30 nm with a maximum at 15 nm. The effect of clustering of the nanoparticles is observed upon drying of the colloidal solution. The silver nanoparticle diameters range from 20 to 500 nm with maximum at 80 nm. It can be seen that a few Ag nanoparticles have a relatively larger diameter in the range of a few hundred nanometers. Comparing these two types of nanoparticles, the Au nanoparticles have a smaller average particle size and better particle uniformity. Since the laser parameters used for the generation of the nanoparticles for both Au and Ag are the same, the difference in the size distribution of the generated nanoparticles is not only due to the laser parameters but also several other factors. One factor is the different thickness of the target sample, which leads to the slightly different DI water layer thickness above the target sample surface. The laser ablation rate can be enhanced by the confinement of the water [18]. For the Au target, the water layer thickness was ≈1 mm, while for the Ag target, the water layer thickness was ≈5 mm. For the Au target, the plasma generated during laser ablation is effectively confined by the 1 mm thick water film, leading to an increased plasma pressure and temperature. Thus, a large amount of energetic species are ejected during the laser ablation. The generated nanoparticles remain in the incident laser light path due to the limited mobility of the nanoparticles in water and the limited volume of the beaker. These nanoparticles can absorb the subsequent laser pulse energy. The size of the nanoparticles that absorb the incident laser light decreases because of the laser-induced fragmentation [19–20]. For the Ag target, having a much thicker water layer, the laser ablation rate is reduced because of the absorption of the laser energy by water. In addition, the laser-induced fragmentation is also reduced due to fewer ablated species and less absorbed laser energy. Hence, the size of the laser-generated Ag nanoparticles is larger. Au and Ag have different physical properties, such as the absorption spectrum of the laser light, melting point, boiling point and thermal conductivity, which all can contribute to the size difference.

**Figure 2:**
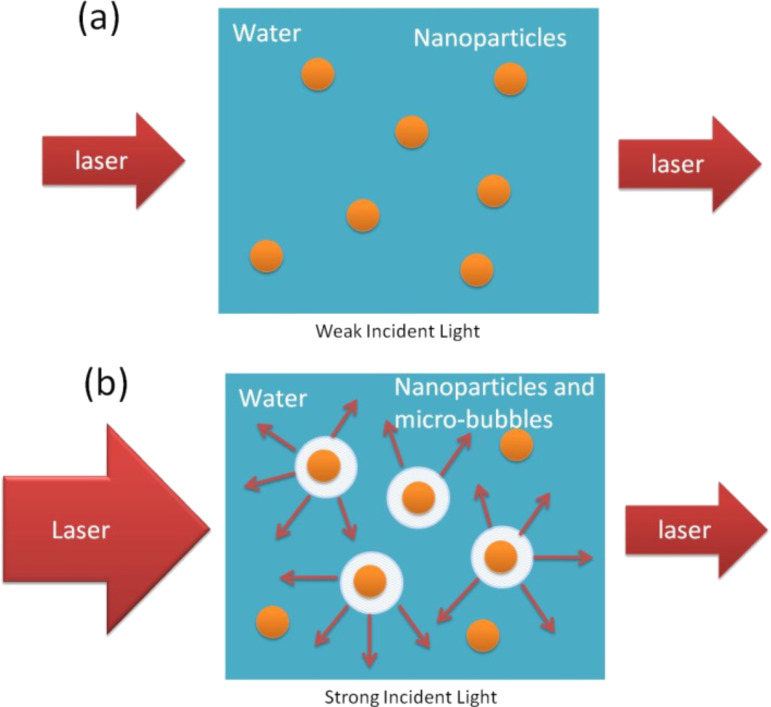
Schematic illustration of the optical limiting effect induced by the nonlinear scattering process. (a) The nanoparticle dispersion is almost transparent upon illumination by laser light at low laser fluence. (b) The optical limiting effect of the nanoparticles appears when microbubbles are formed at high laser fluence.

The laser-generated metallic nanoparticles are promising optical limiting materials. Several mechanisms, as discussed in the Introduction, could be responsible for the optical limiting behavior of these materials. Under the illumination of ns laser pulses at 1064 nm, the optical limiting effect of Au nanoparticles was believed to be due to nonlinear scattering. Polavarapu et al. [21] have shown that nonlinear absorption was not significant for the gold nanoparticles in water. At low laser fluence, the nanoparticle dispersion is almost transparent to the laser light and thus the laser light exhibits a linear transmission through the dispersion as shown in Figure 2a. The optical limiting effect of the nanoparticles appears upon formation of microbubbles. The bubble formation occurs at the solvent–nanoparticle interface. The absorbed photon energy by the nanoparticle is dissipated as heat to the surrounding solvent, leading to solvent vaporization and bubble formation. These bubbles act as scattering centers, which induces the optical limiting effect [22]. Typically, the contribution from nonlinear scattering becomes dominant at high laser fluence as illustrated in Figure 2b.

**Figure 3:**
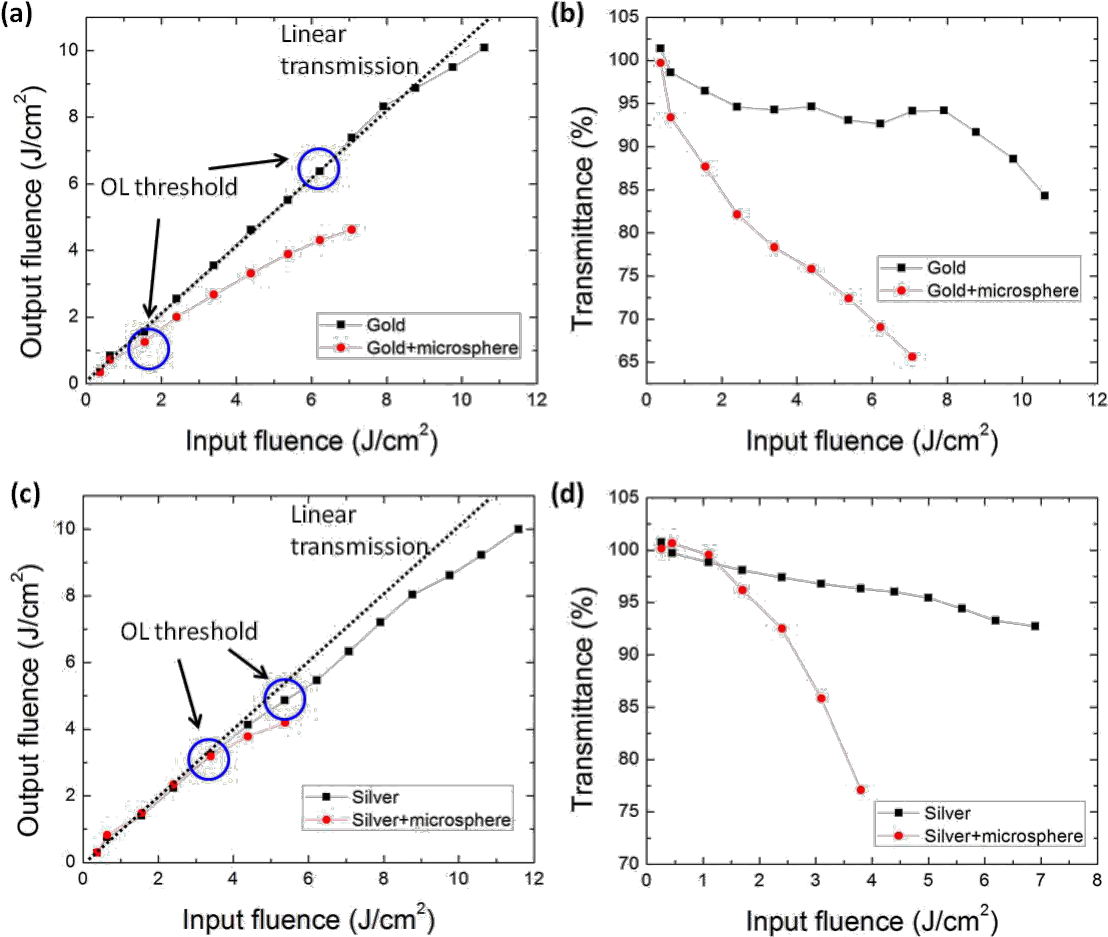
Optical limiting (OL) response and normalized transmittance curves of (a,b) gold nanoparticles and gold/silica nanocomposites and (c,d) silver nanoparticles and silver/silica nanocomposites.

To verify the optical limiting performance of the laser-generated nanoparticles, the nonlinear transmission of these nanoparticles was investigated using a fiber laser operated at a wavelength of 1064 nm and a pulse duration of ≈10 ns. Figure 3 shows the optical limiting performance and the nonlinear transmission results. Both materials (Au and Ag) exhibited a significant optical limiting effect. The threshold for the optical limiting effect is defined as the laser pulse fluence at which the bubble starts to form, causing significant nonlinear scattering and fluctuation in the output power measurement, which is due to the movement of the bubbles. Figure 3a shows the optical limiting response for the gold nanoparticle and the gold/silica nanocomposite dispersions. For the gold nanoparticle dispersion, the optical limiting threshold is about 6.2 J/cm^2^. For the gold/silica nanocomposite dispersion, the optical limiting effect threshold is about 1.6 J/cm^2^. Hence, by adding the silica microspheres, the optical limiting threshold is lowered by 4.6 J/cm^2^. Figure 3b shows the normalized nonlinear transmittance of the samples (gold nanoparticles and gold/silica nanocomposites) upon excitation with ns laser pulses at 1064 nm. The input laser fluence was varied from 0.3 to 10.6 J/cm^2^. As the input fluence is low, the transmission does not change much with input fluence. When the input fluence is further increased, the transmission of all samples starts to decrease. It is clear that the transmittance reduces much more rapidly for the gold/silica nanocomposites than for the pure gold nanoparticle dispersion. Figure 3c,d shows the optical limiting response and normalized transmittance curves of silver nanoparticles and silver/silica nanocomposites. By adding the silica microspheres, the optical limiting threshold is lowered from 5.4 to 3.4 J/cm^2^. The transmittance curves in Figure 3d also show that the optical limiting property of the silver nanoparticles can be improved by addition of silica microspheres. From these results, it can be demonstrated that the optical limiting threshold can be significantly lowered with the addition of silica microspheres.

**Figure 4:**
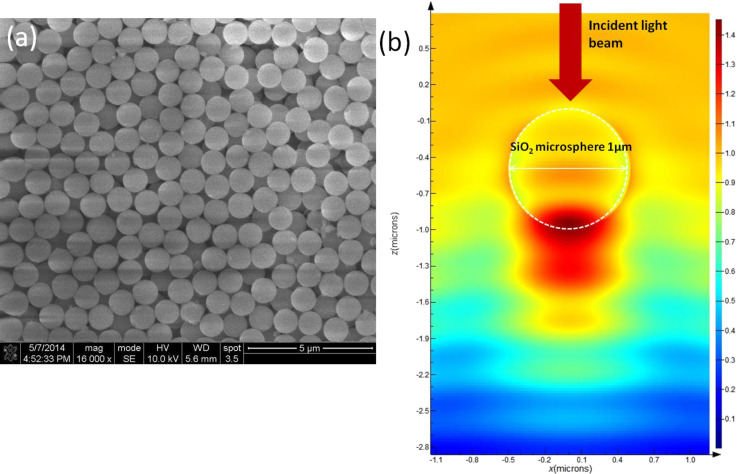
(a) SEM image of silica microspheres and (b) FDTD simulation result for a silica microsphere with a diameter of 1 µm under 1064 nm laser irradiation.

Silica microspheres are known to induce an enhanced local field used for various applications [23–24]. Figure 4a provides a SEM image of the silica microspheres. It shows that the microspheres have uniform shape and size of 1 µm. Figure 4b shows the simulation result using the finite-difference time-domain (FDTD) method with the Lumerical software for predicting the enhancement of the optical nonlinearity by the microspheres. The refractive index of the environment (water) was set at 1.33, while the refractive index of the silica microsphere was set at 1.51. It is observed that the light energy in the area near the silica microsphere is enhanced due to its focusing effect. The enhancement of the light energy is ≈1.4 times higher than the incident light at the center of the enhanced area. The enhancement of the optical nonlinearity is attributed to the local field effect induced by the light focusing of the silica microspheres. We have also simulated the light energy enhancement for different sizes of microspheres, as shown in Table 1. It is found that larger sized microspheres induce a stronger local field enhancement. In our experiment, we found that large sized (>1 μm) microspheres were not stable in the nanoparticle dispersion. The microspheres settle to the bottom of the cuvette after tens of seconds after introducing them in the dispersion. Since the local field enhancement can be increased by larger sized microspheres, high viscosity liquids need to be used instead of DI water to improve the stability of the large sizes microspheres in the dispersion. This will be investigated in future research.

**Table 1:** Field enhancement by microspheres of different sizes.

Microsphere size (μm)	Field enhancement factor

0.5	1.2
1	1.4
1.5	2.2
2	2.8
5	5.9

The introduction of silica microspheres in a metallic nanoparticle dispersion concentrate the light energy into a smaller spot. Such a process causes strong light scattering or absorption, and thus lowers the optical limiting system’s energy threshold. The concentration of light by the transparent microspheres is not limited to a specific wavelength, but rather over a broadband spectral range, making this hybrid optical limiter more compatible for practical applications. Since the microspheres are transparent in the wavelength range of interest, there will be no visual loss to limit the functionalities. Our optical limiting results have shown that silica microspheres are a promising material to enhance the optical limiting effect. In addition, metallic nanoparticles exhibit localized surface plasmon resonance (LSPR), which is another possible way for local field enhancement to influence the light absorption and scattering [25].

## Conclusion

In this paper, we have studied that LAL is a promising technique to generate nanoparticles for various target materials. These nanoparticles exhibit a strong optical limiting effect. It was found that the optical limiting effect of the nanoparticles can be significantly enhanced by adding silica microspheres. The enhanced optical limiting behavior of the hybrid material is attributed to the interaction between the laser-generated nanoparticles and the silica microspheres, when the metal nanoparticles enter the focal volume of the microspheres. The silica microspheres focus the laser light energy onto the metal nanoparticles, which in turn enhances the optical limiting effect of these nanoparticles.
